# Biomarkers for glioblastoma multiforme: status quo

**Published:** 2016-03-27

**Authors:** Nicola Montano, Quintino Giorgio D’Alessandris, Alessandro Izzo, Eduardo Fernandez, Roberto Pallini

**Affiliations:** Institute of Neurosurgery, Catholic University, Rome

**Keywords:** glioblastoma multiforme, prognosis, molecular marker, survival, O(6)-methylguanine-DNA methyltransferase, epidermal growth factor receptor, epidermal growth factor receptor variant III, phosphatase and tensin homolog deletion, vascular endothelial growth factor

## Abstract

**Background:**

Glioblastoma (GBM) is the most frequent and most malignant central nervous system (CNS) tumor. GBM shows poor prognosis with a median overall survival of 14.6 months, despite current surgical and adjuvant therapies. O(6)-methylguanine-DNA methyltransferase (MGMT) methylation is the strongest molecular prognosticator for GBM with therapeutic implications in adjuvant treatment. Isocitrate dehydrogenase (IDH) mutation is the most recently introduced molecular marker and is important for the GBM classification because distinguishes primary (de novo) from secondary GBM. In the last two decades huge advances in the understanding of biopathological bases of gliomagenesis have been made but, to date, there is a lack of biopathological markers endowed of some prognostic and predictive value for GBM.

**Aim:**

In the present review we analyzed the role, as possible prognosticators, of epidermal growth factor receptor (EGFR) variant III (EGFRvIII), phosphatase and tensin homolog (PTEN) deletion and other alteration of the receptor tyrosine kinase (RTK) pathway, and vascular endothelial growth factor (VEGF) expression. We included in the review studies considering both the prognostic value and the predictive value for response to therapy of the above-mentioned biomarkers.

**Relevance for patients:**

These factors have a paramount importance in gliomagenesis and are potential targets for individualized therapies. EGFR can be targeted by tyrosine kinase inhibitors (TKIs). mTOR, whose activation is triggered by PTEN loss, is the target of rapalogs and VEGF is the target of the molecular antibody bevacizumab. Unfortunately, current evidence is insufficient to draw a definite prognostic/predictive role for these biomarkers in GBM. Further understanding the gliomagenesis pathways and looking for biomarkers endowed with translational relevance are necessary efforts in order to find the appropriate, tailored therapy for each specific GBM patient.

## 1. Introduction

Glioblastoma (GBM) is the most frequent and most malignant central nervous system (CNS) tumor [1]. In the USA, GBM incidence is 3.9 cases/100000/year, representing 65-70% of astrocytic tumors and 12-15% of CNS neoplasms with a classical incidence peak between 55 and 74 years [2]. Prognosis of GBM is currently dismal, with a median survival from diagnosis of 14.6 months [3]. Standard-of-care is surgery, intended as maximal safe resection [4], followed by conformal radiotherapy and temozolomide (TMZ) given concurrently to radiotherapy and then as adjuvant therapy for at least 6 cycles [3]. Recent studies evaluating the role of other drugs, particularly bevacizumab, in the management of GBM, failed to establish a new standard-of-care [5,6]. Moreover, upon recurrence, no evidence on the best treatment choice exists [7,8]. The 2007 World Health Organization (WHO) classification of CNS tumors [1] failed to incorporate molecular markers for GBM classification purposes. The upcoming 2016 WHO classification of CNS tumors will likely take into account molecular prognosticators in a renowned “integrated diagnosis” [9]. In detail, the roles of O(6)-methylguanine-DNA methyltransferase (MGMT) promoter methylation and isocitrate dehydrogenase (IDH) mutation are expected to be considered for GBM.

MGMT methylation is currently the strongest molecular prognosticator for GBM [10], though some doubts on its reliability in the first 6 months after GBM diagnosis have been raised [11]. MGMT promoter methylation carries therapeutic implications in adjuvant treatment of GBM in elderly [4]. IDH mutation is the most recently introduced molecular marker for astrocytic tumors, and is considered the first event in multistep gliomagenesis [12]. Therefore, IDH mutation distinguishes primary (de novo) from secondary GBM [13].

In the last two decades huge advances in the understanding of biopathological bases of gliomagenesis have been made and have unveiled a high grade of variability among different GBMs and also among different zones of the same tumor. Thus, GBM is considered as the archetype of heterogeneous cancer [13]. Nonetheless, despite the improvement of knowledge of gliomagenesis, there is a lack of biopathological markers endowed of some prognostic and predictive value. The ideal biomarker in GBM should be easy detectable by routine pathological techniques and highly reproducible among different laboratories and observers. Immunohistochemistry is a reliable, quick, no expensive and widely available technique, but sometimes the more accurate semiquantitative RT-PCR should be preferred. Moreover, an ideal biomarker should clearly identify patients with longer or shorter survival (prognostic role) and/or patients that can benefit from a particular treatment (predictive role). The above-mentioned markers (MGMT and IDH) have an established prognostic/predictive role in GBM and will not be discussed in further detail.

In the present review we will analyze the role as possible prognosticators, of epidermal growth factor receptor (EGFR) variant III (EGFRvIII), phosphatase and tensin homolog (PTEN) deletion and other alteration of the receptor tyrosine kinase (RTK) pathway, and vascular endothelial growth factor (VEGF) expression. At our Institution, EGFRvIII expression, PTEN/mTOR status and VEGF expression have been routinely studied for a decade [8,14,15] not only because they have a paramount importance in gliomagenesis, but also for their role as potential targets for individualized therapies. More specifically, EGFR can be targeted by tyrosine kinase inhibitors (TKIs). It has been showed that EGFRvIII expression is a predictive biomarker for response to TKIs, particularly erlotinib [8,16]. mTOR, whose activation is triggered by PTEN loss, is the target of rapalogs; finally, VEGF is the target of the molecular antibody bevacizumab[5,6]. There has been much enthusiasm in the past years regarding these “targeted therapies”, but results of clinical trials have been rather disappointing. Currently, bevacizumab remains a therapeutic option in recurrent GBM in the USA, whereas erlotinib and rapalogs are experimental drugs whose effectiveness has not been confirmed in unselected cohorts of patients. These evidences foster the search for biomarkers that can identify subgroups of patients who can benefit from targeted therapies [7].

## 2. Biomarkers

Both EGFR and PTEN belong to the RTKs pathway, whose amplification or activation is the hallmark of primary GBM [17]. EGFR is a transmembrane receptor whose activation and dimerization triggers the phosphatidylinositol-3'-kinase (PIP3)-Akt-mammalian target of rapamycin (mTOR) cascade. This pathway plays a crucial role in cellular survival and apoptosis resistance [18]. PTEN is a tyrosine-phosphatase whose role is the inhibition of RTKs pathway [17]. EGFR amplification is the most common molecular alteration in primary GBM [16]. About two thirds of EGFR-amplified GBMs express EGFRvIII, a constitutively-activated, ligand-independent mutant form of EGFR [19]. PTEN mutation has been detected in 15-40% of primary GBM [17]. GBM is, by definition, a high vascular tumor [1] and therefore angiogenesis is a key mechanism for its maintenance and progression [20]. VEGF is the main regulator of angiogenesis in GBM; its production is triggered by tumor hypoxia and it is also released by GBM cancer stem cells in the “vascular niche” [20]. Moreover, VEGF exists in several isoforms with different biological properties [14].

## 3. Search Strategy

A search was performed in the PubMed database (http://www.ncbi.nlm.nih.gov/pubmed/) in February 2016, using the following key words, “glioblastoma”, “EGFRvIII”, “epidermal growth factor receptor variant III”, “VEGF”, “PTEN”, “mTOR”, “Akt”. Articles published after 2005, i.e. after radiochemotherapy with TMZ became the standard-of-care for GBM [3] were included in this review. Review articles as well as redundant publications were excluded from this review.

## 4. Results

### 4.1. Epidermal growth factor receptor variant III

Studies reporting the prognostic or predictive value of EGFRvIII are listed in Table 1[8,15,16,21-34]. We found 17 studies considering 1656 GBM patients overall. The main techniques for assessment of EGFRvIII expression were immunohistochemistry (12/17 studies, 70.6%) and PCR (8/17 studies, 47.1%). Two out of 17 (11.8%) studies (156 patients) reported a positive prognostic value of EGFRvIII expression. Three (17.6%) studies (740 patients) reported a negative prognostic value of EGFRvIII expression. Two (11.8%) studies (60 patients) reported a positive predictive value of EGFRvIII expression for response to TKIs. One (5.9%) study (49 patients) reported a negative predictive value of EGFRvIII expression for response to TKIs. Nine out of 17 (52.9%) studies (651 patients) reported no prognostic/predictive value of EGFRvIII expression.

### 4.2. Phosphatase and tensin homolog and other members of receptor tyrosine kinase pathway

Studies reporting the prognostic/predictive value of PTEN and of other members of RTK pathway are listed in Table 2 [15,16,26,28,29,35-45]. Overall, 16 studies enrolling 1927 patients could be found. PTEN expression was usually detected by immunohistochemistry (11/16 studies, 68.9%). PTEN expression had a positive prognostic value in 5/16 (31.3%) studies (285 patients), whereas no studies in which PTEN loss had a positive prognostic value were found. In 2/5 (40%) studies (98 patients) in which a positive prognostic role of PTEN expression could be demonstrated, iperactivation of EGFR pathway played a synergistic role. In one out of 16 (6.3%) studies (50 patients), PTEN expression had a positive predictive value for response to TKIs. In one other study (32 patients), a positive prognostic value of p-Akt expression was detected. Finally, in 9 out of 16 (56.3%) studies (1560 patients), no prognostic/predictive value of PTEN expression could be determined.

**Table 1.** Studies evaluating prognostic and/or predictive role of EGFRvIII in GBM

***Author, year***	No. GBM cases	Treatment	Assay Technique	Prognostic/predictive value	Proposed molecular mechanism
Heimberger et al, 2005 [21]	196	Stupp	IHC	↓ (for long survivors)	cell proliferation, ependymal involvement
Liu et al, 2005 [22]	160	Stupp	PCR	none	NA
Heimberger et al, 2005 [23]	54	Stupp	IHC	none	NA
Mellinghoff et al, 2005 [16]	50	erlotinib / gefitinib (recurrence)	IHC, PCR, western blotting	↑ (erlotinib)	EGFRvIII/PTEN co-expression
Pelloski et al, 2007 [24]	509	Stupp	IHC	↓ (OS)	none
Viana-Pereira et al, 2008 [25]	27	Stupp	IHC	none	NA
Brown et al, 2008 [26]	81	Stupp + erlotinib	IHC	none	NA
Van den Bent et al, 2009 [27]	49	erlotinib vs TMZ/BCNU	IHC	↓ (erlotinib)	none
Thiessen et al, 2009 [28]	16	lapatinib (recurrence)	PCR	none	NA
Reardon et al, 2009 [29]	20	erlotinib + sirolimus (recurrence)	IHC	none	NA
Uhm et al, 2011 [30]	96	RT+ gefitinib	IHC	none	NA
Montano et al, 2011 [15]	73	Stupp	PCR	↑ (OS)	increased effectiveness of TMZ in EGFRvIII-positive cells due to pathway addiction
Lv et al, 2012 [31]	35	cetuximab	IHC, PCR	↓ (OS, PFS)	none
Bienkowski et al, 2013 [32]	83	RT or RT+CT	PCR, FISH	↑ (OS)	none
D’Alessandris et al, 2013 [8]	10	bev + erlotinib (recurrence)	PCR	↑ (erlotinib)	tailored therapy
Weller et al, 2014 [33]	184	Stupp	IHC, PCR, MLPA	none	possible false-negative testings
Gallego et al, 2014 [34]	13 recurrent	erlotinib	IHC	none	genetic heterogeneity of GBM

↓ negative prognostic/predictive value;

↑ positive prognostic/predictive value.

bev, bevacizumab; BCNU, bis-chloroethylnitrosourea; CT, chemotherapy; EGFRvIII, epidermal growth factor receptor variant III; FISH, fluorescence in situ hybridization; IHC, immunohistochemistry; MLPA, multiplex ligation-dependent probe amplification; NA, not available; OS, overall survival; PCR, polymerase chain reaction; PTEN, phosphatase and tensin homolog; PFS, progression-free survival; RT, radiotherapy; TMZ, temozolomide.

### 4.3. Vascular endothelial growth factor

Details on 11 studies (445 patients) analyzing the prognostic/predictive value of VEGF expression are given in Table 3 [8,14,46-54]. VEGF expression was mostly analyzed by immunohistochemistry (7/11 studies, 63.6%). Overexpression of VEGF had a positive prognostic value in one out of 11 (9.1%) studies (27 patients) and a negative prognostic value in 3/11 (27.3) studies (183 patients). Overexpression of VEGF was predictive of response to anti-angiogenic therapy in 2/11 (18.2%) studies (37 patients). Circulating VEGF levels carried a negative prognostic value in one study (47 patients). One study on 25 GBM patients assessed the predictive value of tumor VEGF isoforms for response to anti-angiogenic therapy. Finally, in 3/17 (27.3%) studies (126 patients) VEGF expression had no prognostic/predictive value.

## 5. Discussion

The aim of this review was to perform a thorough, up-to-date review of prognostic and predictive value of EGFRvIII expression, PTEN deletion and other alteration of the RTK pathway, and VEGF expression. We decided to focus our work on these markers because of their importance in gliomagenesis, their role as potential targets for individualized therapies and because of their translational relevance is currently under debate. EGFRvIII is a constitutively activated mutant form of EGFR. Traditionally, EGFRvIII has been considered an oncogenic protein [19], due to preclinical studies in which EGFRvIII-expressing cells had a superior invasiveness, proliferation and tumorigenesis potential [55,56]. The majority of studies listed in Table 1 are in accordance with this assumption. Nonetheless, in these studies EGFRvIII expression was studied mainly by immunohistochemistry, whose reliability as assay method has been showed inferior to PCR [57]. Interestingly, the studies which demonstrated a positive prognostic and a predictive role for response to TKIs of EGFRvIII adopted the semiquantitative PCR determination as assay method. This discrepancy could be explained by the fact that very low levels of EGFRvIII can be detected by PCR as opposed to immunohistochemistry where the specimens would likely be determined as negative. As concerns PTEN, there is a general agreement on the positive prognostic role of its retained expression. Of note, a tight interaction of PTEN and EGFR expression is documented in several studies. Taken cumulatively, studies listed in Table 1 and Table 2 appear in line with the pioneering study by Mellinghoff *et al*. [16], in which co-expression of EGFRvIII and PTEN had a positive predictive value to response to TKIs. However, this hypothesis probably deserves further validation, at least in the setting of recurrent GBM [15]. Overexpression of VEGF appears to carry a negative prognostic value in term of survival but a positive predictive value to response to anti-angiogenic therapy. Unfortunately, this evidence has been insufficiently tested in the large phase III trials with bevacizumab, conducted on newly diagnosed GBM [5,6]. Interestingly, Kesari *et al*. [49] evaluated the prognostic role of serum VEGF levels. This kind of assay is widely used in other tumors [58], but evidence regarding GBM is low. Another promising research field, which has been pioneered by our group, is the analysis of the role of VEGF isoforms [14], which originate from alterative splicing of the VEGF gene and have different biological properties [59]. In detail, the heavier isoforms (VEGF-206, VEGF-189) are bound to extracellular matrix and act both as VEGF reserve and as trigger of local angiogenesis. The lighter VEGF-121 is freely diffusible but has a weaker biological activity; the intermediate-weight VEGF-165 has also intermediate properties. Consistently with these premises, it has been showed that patients with high level of VEGF-121 responded poorly to bevacizumab, probably because in these patients a lesser residual amount of bevacizumab is available to target the heavier and more active VEGF isoforms [14].

**Table 2.** Studies evaluating prognostic and/or predictive role of PTEN/Akt/mTOR pathway in GBM

Author, year	No. GBM cases	Treatment	Assay Technique	Prognostic/predictive value	Proposed molecular mechanism
Mellinghoff et al, 2005 [16]	49	Erlotinib/gefitinib (recurrence)	PCR, IHC	PTEN: ↑ (for erlotinib)	EGFRvIII/PTEN co-expression
Ohgaki and Kleihues, 2005 [35]	680	Stupp	NA	PTEN: none	NA
Rich et al, 2005 [36]	41	Stupp	PCR, microarray	PTEN: none	NA
Liu et al, 2006 [37]	25	NA	IHC	PTEN loss+ EGFR amplification: ↓ (OS)	none
Homma et al, 2006 [38]	420	Stupp	PCR	PTEN: none	NA
Fukushima et al, 2006 [39]	63	Stupp	SSCP, sequencing	PTEN: none	NA
De Groot et al, 2008 [40]	43	Carboplatin + erlotinib (for recurrence)	IHC	PTEN: none	NA
Brown et al, 2008 [26]	81	Stupp +Erlotinib	IHC	PTEN: none	NA
Thiessen et al, 2010 [28]	16	Lapatinib (recurrence)	IHC	PTEN: none	NA
Umesh et al, 2009 [41]	54	Stupp	IHC	PTEN loss: ↓ (OS)	associated EGFR expression
Ruano et al, 2009 [42]	194	Stupp	IHC	PTEN: none	NA
Kreisl et al, 2009 [43]	22	Gefitinib + everolimus (recurrence)	IHC	PTEN: none	NA
Reardon et al, 2010 [29]	32	Erlotinib+ Sirolimus (recurrence)	IHC	p-AKT +: ↑ (PFS)	unclear. Possible laboratory errors
Montano et al, 2011 [15]	73	Stupp	IHC	PTEN normal + EGFRvIII positive: ↑ (OS)	tumor suppression role of PTEN
Srividya et al, 2013 [44]	73	Stupp	FISH for homozygous deletion of 10q23*/PTEN*	PTEN loss: ↓ (OS)	Tumor suppression role of PTEN
Idoate et al, 2014 [45]	60	Stupp	IHC, PCR	PTEN loss: ↓ (OS)	Tumor suppression role of PTEN

↓ negative prognostic/predictive value;

↑ positive prognostic/predictive value.

EGFR, epidermal growth factor receptor; EGFRvIII, EGFR variant III; FISH, fluorescence in situ hybridization; IHC, immunohistochemistry; NA, not available; OS, overall survival; PCR, polymerase chain reaction; PFS, progression-free survival; PTEN, phosphatase and tensin homolog; SSCP, single-strand conformation polymorphism.

**Table 3.** Studies evaluating prognostic and/or predictive role of VEGF in GBM

Author, year	No. GBM cases	Treatment	Assay Technique	Prognostic/predictive value	Proposed molecular mechanism
Tuettenberg et al, 2005 [46]	12	Stupp + rofecoxib	IHC	none	NA
Pope et al, 2008 [47]	52	Stupp	micro-array	none	NA
Flynn et al, 2008 [48]	62	Stupp	IHC	Overexpression: ↓ (for OS)	GLUT-1 coexpression, hypoxia
Kesari et al, 2008 [49]	47	Stupp + thalidomide and celecoxib	ELISA	High serum levels: ↓ (for OS)	none
Sathornsumetee et al, 2008 [50]	27	bev + CPT-11 (recurrence)	IHC	Overexpression: ↑ (for response)	targeted therapy
Reardon et al, 2009 [51]	27	bev + etoposide (recurrence)	IHC	Overexpression: ↑ (for OS)	targeted therapy
Sie et al, 2009 [52]	62	Stupp	IHC	none	NA
D’Alessandris et al, 2013 [8]	10	bev + erlotinib (recurrence)	IHC	overexpression: ↑ (for response)	tailored therapy
D’Alessandris et al, 2015 [14]	25	bev (recurrence)	PCR	total VEGF and VEGF-121: ↓ (for PFS)	Heavier VEGF isoforms are the main target of bev
Irshad et al, 2015 [53]	35	NA	PCR	activation of hypoxia cascade (incl. VEGF): ↓ (for OS)	Oncogenic role of hypoxia
Zhao et al 2016 [54]	86	Stupp	IHC	VEGF-C overexpression: ↓ (for OS)	VEGF-C stimulates NRP2 in paracrine/ autocrine loop

↓ negative prognostic/predictive value;

↑ positive prognostic/predictive value.

bev, bevacizumab; CPT-11, irinotecan; ELISA, enzyme linked immunosorbent assay; IHC, immunohistochemistry; NA, not available; NRP2, neuropilin-2; OS, overall survival; PCR, polymerase chain reaction; PFS, progression-free survival; RT, radiotherapy; TMZ, temozolomide; VEGF, vascular endothelial growth factor.

Taken altogether, the results of our study show that none of the markers analyzed in this review reached sufficient evidence to be considered a clear prognosticator for survival. Moreover none of these potential biomarkers was clearly predictive of response to adjuvant therapy. This data is particularly unpleasant in the setting of recurrent GBM, in which there is a strong need of predictive biomarkers for response to targeted therapy [7]. The number of studies in which no role for biomarkers could be detected is also troublesome; this can reflect both GBM heterogeneity and the inaccuracy of the assay methods employed.

In order to improve biomarkers detection and validation, current and future clinical trials need to prospectively assess their potential role. Several subgroups should be designed, with the aim of tailoring the treatment on patient’s molecular profile. This claims for large, collaborative, multicenter trials, able to reach adequate recruitment targets. In order to minimize biomarkers determination errors, a few pathology “reference” laboratories for each country should be identified that intratumor heterogeneity can be solved by analyzing several tumor samples from different regions. When applicable, analysis of cancer stem cells profile can provide a reliable picture of the tumor’s landscape [60]. A new and intriguing opportunity for studying the genomic and/or proteomic profile of GBM for prognostic or predictive purposes is provided by the so-called “liquid biopsies”, i.e. the analysis of peripheral blood samples. The main target of these biopsies are the circulating tumor cells, which can be analyzed using standard immunocytochemical or molecular biology techniques. Liquid biopsies are currently used for prognostic/predictive purposes in several tumors. In GBM, the difficulty to identify affordable biomarkers to separate circulating tumor cells from normal blood cells [61] has hindered the development of this technique. Alternatives to circulating tumor cells, to be analyzed in a liquid biopsy, include RNA sequencing of “tumor-educated platelets” [62] and genomic profiling of microvescicles [63].

Understanding the gliomagenesis pathways and looking for biomarkers endowed with translational relevance are hard tasks in a heterogeneous tumor like GBM. However, this is a necessary effort in order to find the appropriate, tailored therapy for each specific GBM patient.
